# A case report of an unusual mycotic pseudoaneurysm of the ascending aorta

**DOI:** 10.1186/s13019-022-01989-2

**Published:** 2022-09-22

**Authors:** Tobin P. Mangel, Damian Balmforth, Aung Oo

**Affiliations:** Department of Cardiothoracic Surgery, St Bartholomew’s Hospital, W Smithfield, London, EC1A 7BE UK

**Keywords:** Mycotic, Pseudoaneurysm, Aorta

## Abstract

**Background:**

Mycotic pseudoaneurysms of the ascending aorta are a rare and devastating complication of previous cardiac surgery.

**Case presentation:**

We present an unusual case of a fungal mycotic pseudoaneurysm secondary to an aortic suture line successfully repaired under deep hypothermic circulatory arrest.

**Conclusions:**

Patients with mycotic pseudoaneurysms of the aorta require a multidisciplinary team approach to prevent devastating complications that may occur in these complex surgical cases.

## Background

**Fig. 1:**
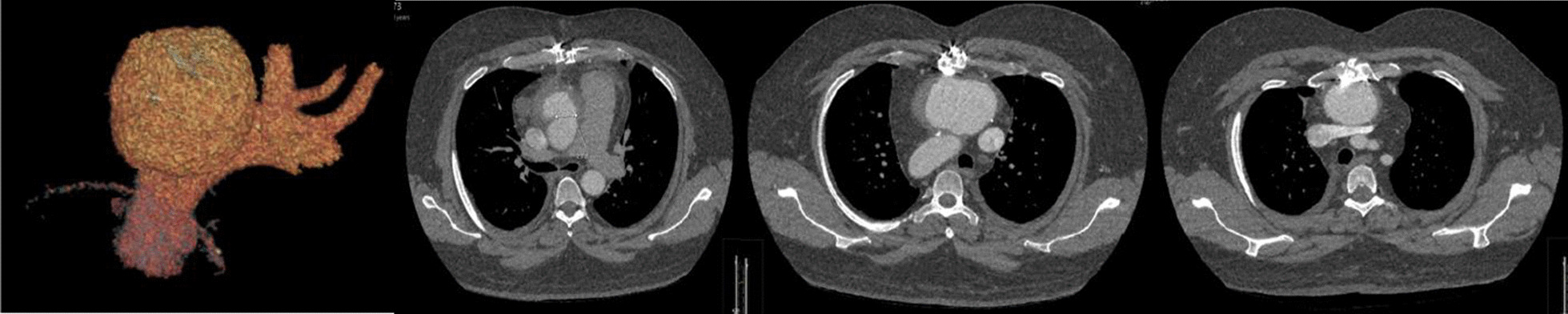
Pre-operative imaging

**Fig. 2:**
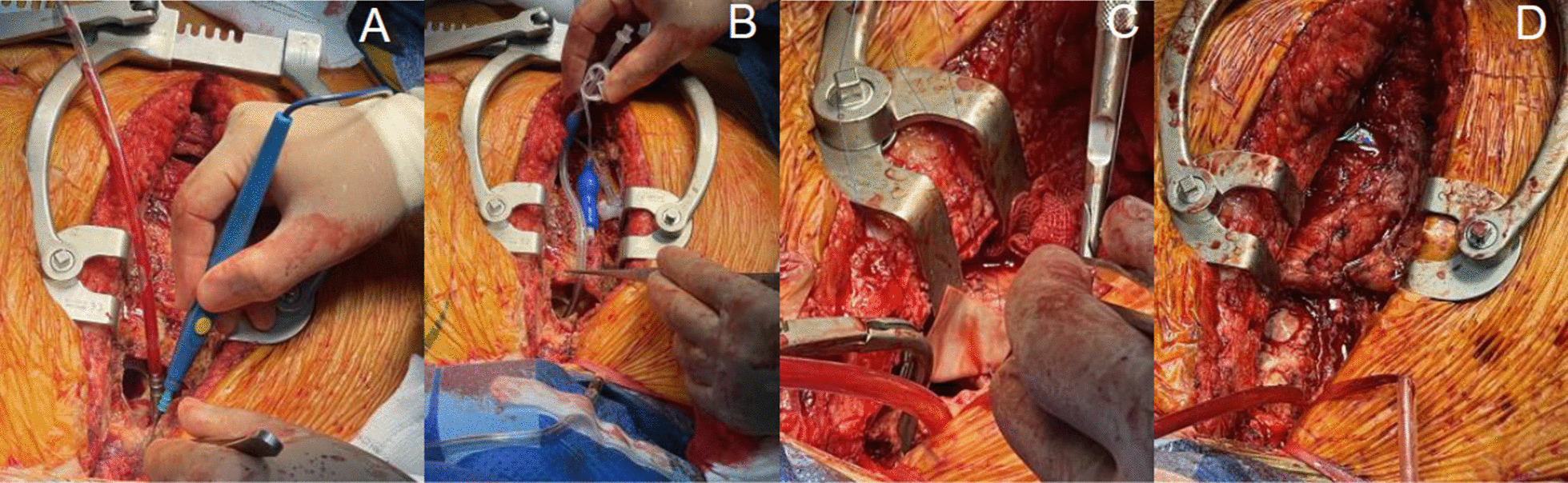
**A** Opening aneurysm sac. **B** Retrograde cannula inserted to occlude defect to allow aorta to be dissected and cross clamp to be applied. **C** Patch repair under cross clamp. **D** Patch repair completed and cross clamp off

Mycotic pseudoaneurysms of the ascending aorta are rare, particularly those related to fungal organisms such as Aspergillus. We present an unusual case of a mycotic pseudoaneurysm secondary to an unroofing procedure for anomalous aortic origin of a coronary artery (AAOCA) and discuss the potential treatment options. (Figs. 1 and 2).

### Case presentation

A 48 year old gentleman initially presented with chest pain and exertional dyspnoea and was found to have an aberrant right coronary artery (RCA) arising from the left coronary sinus. He underwent an unroofing procedure to re-site the origin of the right coronary artery in the correct sinus. His recovery was uncomplicated with no signs of infection.

Three months post discharge, the patient presented to ophthalmology with an acutely red painful left eye. The patient was admitted for antivirals and steroids for possible herpes simplex virus (HSV) infection, but despite this treatment he had lost his vision in the left eye by July 2021.

In August of 2021, the patient represented with rapidly deteriorating vision in his right eye and was transferred to a tertiary ophthalmology unit. Corneal scrapings demonstrated *Aspergillus Fumigatus and Saccharomyces Cerevisiae* and a CT thorax abdomen and pelvis confirmed disseminated fungal infection and fungal endophthalmitis. A 74 mm × 76 mm saccular pseudoaneurysm was found arising from the mid anterior ascending aorta, with a wide 24 × 21 mm diameter orifice and no neck. The pseudoaneurysm was in close apposition to the manubrium and proximal sternal body. The features were in keeping with a large fungating mycotic pseudoaneurysm arising from the previous aortic suture line and aspergillus infection was strongly suspected. The patient was commenced on anti-mycotic therapy and transferred to a quaternary aortovascular centre for re-sternotomy and repair of the ascending aortic pseudoaneurysm.

The patient was placed on cardiopulmonary bypass via the right axillary artery and right femoral vein. During cooling a left anterior thoracotomy was performed in the 5th intercostal space and a left ventricular vent sited via the apex of the heart. Once a core temperature of 24 degrees was reached, the circulation was arrested prior to resternotomy. On opening the chest, the aneurysm sac had a fungating appearance and breached. A retrograde cardioplegia cannula was placed through the defect in the ascending aorta and the balloon inflated to occlude the opening. The innominate artery was dissected out and snared allowing antegrade cerebral perfusion (ACP) to be delivered via the arterial cannula in the right axillary artery. The distal ascending aorta was then dissected out and a cross-clamp applied allowing the circulation to be restarted after 30 min of deep hypothermic circulatory arrest (DHCA). The infected pseudoaneurysm sac was dissected out and the defect in the ascending aorta closed with a pericardial patch. The patient was admitted to ITU for post-operative monitoring where he made an uncomplicated recovery. He was repatriated back to his local hospital for ongoing ophthalmology care.

## Discussion

Mycotic pseudoaneurysms are often caused *by Staphyloccous, Streptococcus* or *Salmonella* rather than fungal infections, and present with nonspecific systemic sepsis like symptoms [1]. Aspergillus infection is rarely seen in an immunocompetent patient such as in the current case.

Aortic pseudoaneurysms can arise along anastomotic suture lines or cannulation sites following previous cardiac surgery [1]. Treatment options for pseudoaneurysms in the ascending aorta abutting the sternum are limited and depend on pre-empting the probability of entering the aneurysm on resternotomy. The theatre team must be briefed to expect this outcome and strategies put in place to manage it. The strategy utilised in the current case was to open the sternum under DHCA. The left ventricular apical vent was sited to prevent ventricular distension when the heart fibrillates during cooling [2]. We prefer cannulation of the right axillary artery over the right femoral artery due to the reduced risk of organ mal-perfusion or retrograde embolism and the ability to deliver ACP upon snaring the innominate artery [3].

DHCA with antegrade or retrograde cerebral perfusion is a strategy used to optimise cerebral protection and provides a bloodless field during complex aortic operations [4]. There is no consensus on duration and optimal temperature for DHCA with the decision left to the operating surgeon. Moderate hypothermia (> 25C) with ACP is an alternative strategy for aortic surgery and it is proposed to have similar outcomes as DHCA [3]. This strategy was deemed unsuitable for the current case as it was not possible to quickly instigate ACP following commencement of DHCA due to the need to complete the sternotomy and dissect out the adhered pericardial structures. The role of the anaesthesiologist and perfusionist are critical in minimising the risk of cerebral ischaemia during this period of DHCA without cerebral perfusion.

An alternative approach to this scenario of a pseudoaneurysm abutting the sternum would have been to perform trans-femoral aortic endoclamping. This approach takes careful planning, is not appropriate for urgent or emergency cases, requires significant preoperative monitoring, and would not be advisable in patients with pseudoaneurysm near the brachiocephalic trunk [5]. It also risks precipitating a rupture of the pseudoaneurysm by applying a radial force on the aortic wall. As such it was not considered in this case despite the potential benefits of not requiring DHCA, reducing cardiopulmonary bypass time and the risk of hypoxic brain injury.

## Conclusion

In patients who present with thromboembolic events or are systemically unwell after bypass surgery, we urge clinicians to have low threshold for suspicion of pseudoaneurysms secondary to bacterial or fungal infections. Patients with mycotic pseudoaneurysms of the aorta abutting the sternum require a team approach to control for significant blood loss and minimise the risk of cerebral ischaemia that may occur on opening the pseudoaneurysm sac. Case specific planning is essential and ultimately made a potentially complex case, straight forward with a positive outcome.

## Data Availability

Not applicable.

## Abbreviations

AAOCA: Anomalous aortic origin of a coronary artery
RCA: Right coronary artery
HSV: Herpes simplex virus
ACP: Antegrade cerebral perfusion
DHCA: Deep hypothermic circulatory arrest
